# Frontiers in assessing septic systems vulnerability in coastal Georgia, USA: Modeling approach and management implications

**DOI:** 10.1371/journal.pone.0256606

**Published:** 2021-08-25

**Authors:** Nahal Hoghooghi, J. Scott Pippin, Brian K. Meyer, John B. Hodges, Brian P. Bledsoe

**Affiliations:** 1 Institute for Resilient Infrastructure Systems, College of Engineering, University of Georgia, Athens, Georgia, United States of America; 2 Department of Geosciences, Georgia State University, Atlanta, Georgia, United States of America; Universidade Federal de Uberlandia, BRAZIL

## Abstract

Threats to public health and environmental quality from septic systems are more prevalent in areas with poorly draining soils, high water tables, or frequent flooding. Significant research gaps exist in assessing these systems’ vulnerability and evaluating factors associated with higher rates of septic systems replacement and repair. We developed a novel GIS-based framework for assessing septic system vulnerability using a database of known septic system specifications and a modified Soil Topographic Index (STI) that incorporates seasonal high groundwater elevation to assess risks posed to septic systems in coastal Georgia. We tested the hypothesis that both the modified STI and septic system specifications such as tank capacity per bedroom and drainfield type would explain most of the variance in septic system repair and replacement using classification inference tree and generalized logistic regression models. Our modeling results indicate that drainfield type (level vs. mounded) is the most significant variable (*p*-value < 0.001) in predicting septic systems functionality followed by septic tank capacity per bedroom (*p*-value < 0.01). These show the importance of septic system design regulations such as a minimum requirement for horizontal separation distance between the bottom of trenches and seasonal water table, and adequate tank capacity design. However, for septic systems with a mounded drainfield and a larger tank capacity per bedroom, the modified STI representing hydrological characteristics of septic system location is a significant predictor of a high septic system repair and replacement rate. The methodology developed in this study can have important implications for managing existing septic systems and planning for future development in coastal areas, especially in an environment of rapid climatic change.

## Introduction

Septic systems consist of a tank and a soil treatment area or a drainfield [1]. Wastewater flows into the tank where solid material settles to the bottom, and the remaining effluent flows out of the tank into a drainfield where it leaches into the ground. The initial treatment occurs in a septic tank, where most of the settleable and floatable materials are removed and partial digestion of organic matter occurs under anaerobic conditions. Microbes in the soil and other biological processes further breakdown the remaining contaminants to yield treated effluent that delivered to groundwater, and in many instances surface waters [1]. Septic systems, are a widespread component of communities’ wastewater infrastructure throughout the United States, particularly in the eastern states [2]; however these systems are generally viewed as part of individual properties and thus the role they play in communities’ wastewater infrastructure is often unrecognized.

Globally, 4.2 billion people lack safely managed sanitation [3]. Septic systems primarily exist in rural areas, on barrier islands, and in other areas difficult to reach with sewer lines or where sewer lines are not desired by local residents. The US Environmental Protection Agency (USEPA) estimates approximately 24% of homes are served by septic systems nationwide [1], and the US Census Bureau estimates that there are about 22 million households rely on septic systems for their wastewater treatment [4].

According to the USEPA, septic system functionality is defined by the system’s ability to remove settleable solids, nutrients, and pathogens from wastewater discharges [1]. When functioning, septic systems provide adequate treatment of human wastewater and are an integral part of a community’s wastewater infrastructure where implementation of central wastewater systems is infeasible or cannot expand fast enough to serve growing populations [1, 2]. However, public health and environmental quality can be negatively affected when these systems fail. Septic effluent contains potentially dangerous pathogens as well as concentrations of nitrogen, phosphorus, and pathogens can significantly impact environmental quality [5]. Septic systems often contribute excessive nitrogen and phosphorus loads to surface and ground waters, thereby exacerbating eutrophication and harmful algal blooms in freshwaters [6, 7], changes in food web dynamics, and loss of biodiversity and habitat [8–12]. Further, excessive nitrate concentrations in drinking water resulting in methemoglobinemia or “blue baby syndrome” [13] and disease outbreaks (e.g., acute gastroenteritis) associated with poor septic tank performance have been reported globally [14, 15].

No state has directly measured its septic systems failure rate and definitions of failure vary [16]. The most obvious usually involves some form of mechanical or structural failure in the system that results in discharges of untreated effluent on the surface of the ground or the overflow of wastewater in the structure served by the system. This leads to human exposure to waste and potentially significant property damage. These are the types of failures that attract the attention of property owners and regulators and thus result in repairs. Whether caused by structural failure, improper siting, poor construction, lack of maintenance, or changes in environmental conditions, this type of failure generally goes unnoticed, and rarely result in repairs. Therefore, when discussing system failure in this paper, we are considering the former type of failure that result in direct discharges of wastewater into surface or ground waters.

Assessments of septic system failures are very local, generally limited to a single city, county, or neighborhood, and the results vary widely and are not transferable to other regions [16]. There are few general estimates of failure at larger scales, and even state level analyses of septic system repair records use different types of records and varying definitions of failure making comparison difficult. The only general assessment of the frequency of septic system failure identified was a 1997 USEPA reference to a survey conducted by the U.S. Census Bureau that estimated 403,000 homes experienced a septic system breakdown in a three-month period, and the USEPA’s literature review cited failure rate ranging from 1 to 20 percent [1]. This lack of readily available information on septic system functionality is a severe limitation to understanding the current impact of septic systems on public health and water quality, and it makes it impossible to even begin to assess the future impacts climate change will have on the functionality of these systems and thus in the environment and our communities. To better understand the functionality of septic systems, we propose a novel methodology to assess the vulnerability of a septic system to failure.

Significant research gaps exist in assessing septic systems vulnerability and factors associate with higher rate of septic systems replacement and repair. Kohler et al., 2016 assessed the association of “Onsite Wastewater Treatment System (OWTS) fragility” (the degree to which a system loses functionality) with local temperature, rainfall, and streamflow conditions over a range of time scales for 225 septic systems with available frequency of septic system repairs report in Boulder County, Colorado, USA. The results of their generalized linear regression model showed that high temperature, frequency of wetter-than-normal months, and magnitude of peak streamflow in the watershed impact on complete loss of septic system functionality [17]. However, they did not include the groundwater conditions, septic system specifications, and site hydrologic characteristics in their analysis.

Because septic effluent treatment relies on hydrologic, microbial and chemical processes, wastewater treatment in the drainfield area is sensitive to changes in soil moisture [18]. Septic system failure has been shown to be more prevalent in areas with poorly draining soils, high water tables, and frequent flooding. The Topographic Wetness Index (TWI) [19] has been shown to be a good predictor of soil moisture content or shallow groundwater level [20–23], and it had been used to quantify frequencies and durations of saturated soil conditions [24]. Until recently, TWI-based measures had not been widely used in low relief landscapes such as coastal areas, owing to issues with low-resolution Digital Elevation Model (DEM) that are plagued with pits, flat areas, and flow accumulation that may follow vertical errors of the raster. However, TWI-based indices like Soil Topographic Index (STI) have been successfully used in conjunction with high-resolution Light Detection and Ranging (LiDAR) to accurately identify areas of elevated soil moisture and subtle differences of wetland types and boundaries in flat coastal plain environments [25, 26].

In this study, we use a novel application of the STI incorporating groundwater levels to develop methods and create a GIS-based framework for septic system vulnerability. Since many of the conditions associated with system failure are common conditions in coastal areas, making coastal regions problematic for siting and maintaining septic systems [27, 28], we developed this method in coastal Georgia. Specifically, we coupled the baseline seasonal high groundwater elevation with STI [29] to create a modified STI value for all parcels in the southern part of Bryan County, Georgia.

To assess the degree of risks posed to septic systems under current groundwater elevation, we used a newly developed GIS database of recorded septic systems in Bryan County, Georgia in our analysis. This database contains numerous data fields for the septic system attributes. We hypothesize that both the modified STI and septic systems specifications such as tank capacity per bedroom and drainfield type would explain most of the variance in septic systems failure resulting in repair or replacement. We use a classification inference tree and generalized logistic regression models to analyze a binary qualitative variable, like whether a septic system installation is new or replacing an existing system, as a function of a number of expletory variables such as the modified STI, whether the septic drainfield is installed in a mound, and septic tank capacity per bedroom. Based on the results of statistical analyses, we create a map depicting relative risk of septic systems vulnerability in southern Bryan County, Georgia. With a methodology to assess the vulnerability of septic systems to failure, we discuss the policy, planning, and management implications and opportunities presented by the availability of this data.

## Materials and methods

### Study area description

Coastal Georgia remains among the least developed coastlines in the US and is characterized by extensive marshes, estuaries, and barrier islands, many of which remain undeveloped. The success at preserving Georgia’s coastal resources is fueling increasing pressure for residential and commercial growth in the coast region. Bryan County, Georgia was selected for this research project both because they potentially face significant impacts from septic systems impacted by rising sea levels, and their septic data was robust and well maintained, which facilitated its use in this study.

**Fig 1:**
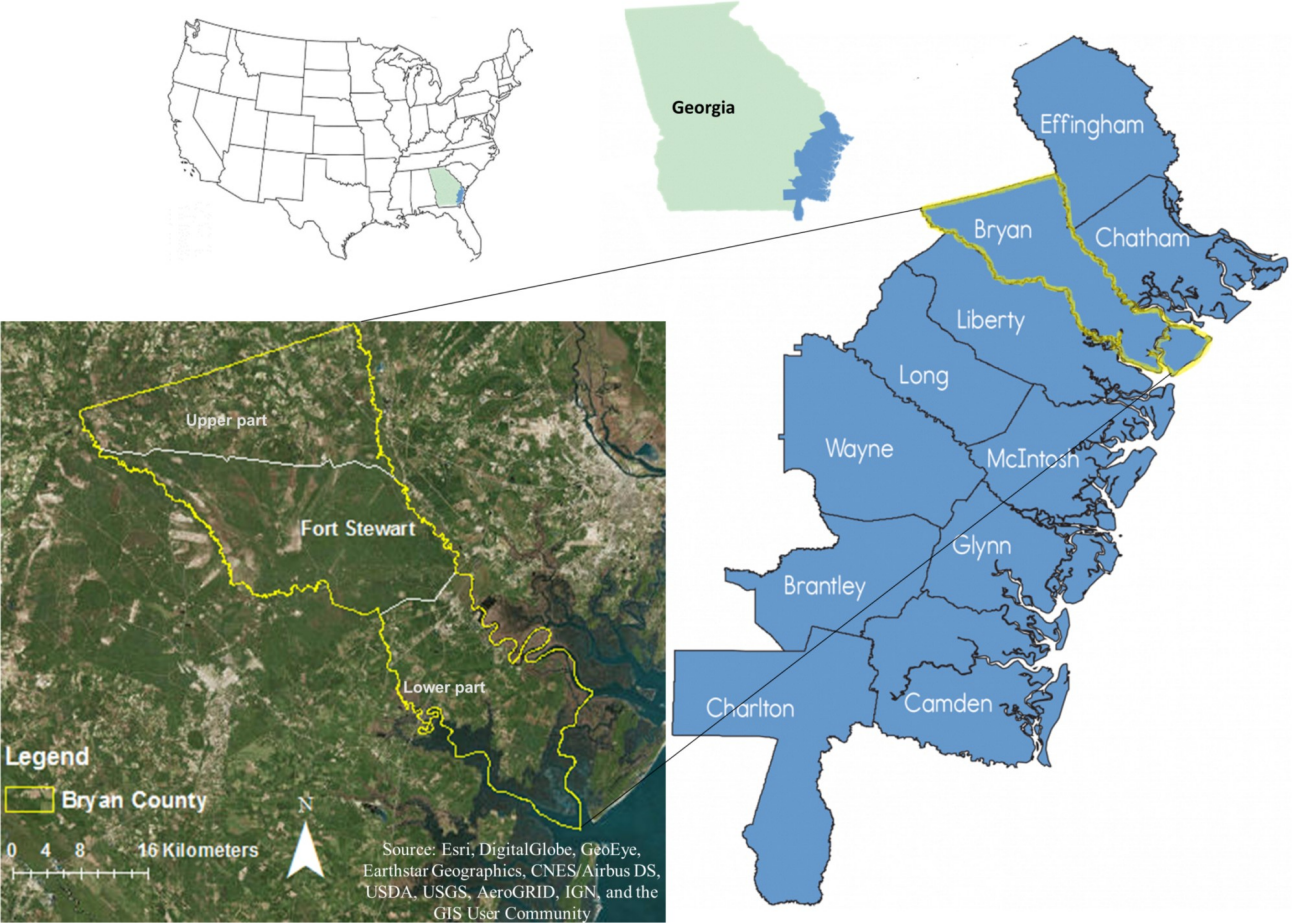
The study area is in the Coastal Plain of Georgia located at the southern part of Bryan County, Georgia, USA. Adopted and modified from Georgia Department of Natural Resources, Coastal Resources Division [32, 33].

Bryan County, Georgia, USA, is located in the Georgia Coastal Plain in the Ogeechee River basin near the center of the Georgia Bight and southwest of Savannah (Fig 1) and has a total area of 1180 Km^2^, with approximately four percent of the total area is water [30]. The weather is subtropical with high humidity, temperature, and average annual precipitation is 1295 mm and air temperature ranges from 9.4 to 27.2°C [31].

Since 2010, Bryan County has been the second fastest growing county in Georgia; the population increased by 31% from 2010, with 30,233 residents to 2019, with 39,627 residents [34]. This population growth has fueled rapid residential development in Bryan County that is primarily based on septic systems. Much of this development is occurring in areas that will be impacted by sea level rise. Moreover, the Bryan County water system gets its water from Floridan aquifer [35]which could be negatively impacted with poor septic systems functioning in the future. The increasing number of septic systems and the fact that future development will likely continue to follow this trend, means that officials in Bryan County have a strong need to understand how these systems will function under changing environmental conditions. At the same time, the fact that most of the county’s septic records have been created in the last 20 years that is more uniform and complete than those in some other counties where a significant proportion of the records are more than 40 or 50 years old. This clean dataset coupled with the collaborative relationship with the local government were important components of this research, and expanding the use of this methodology into areas where these factors are not present is an area for continued work.

**Fig 2:**
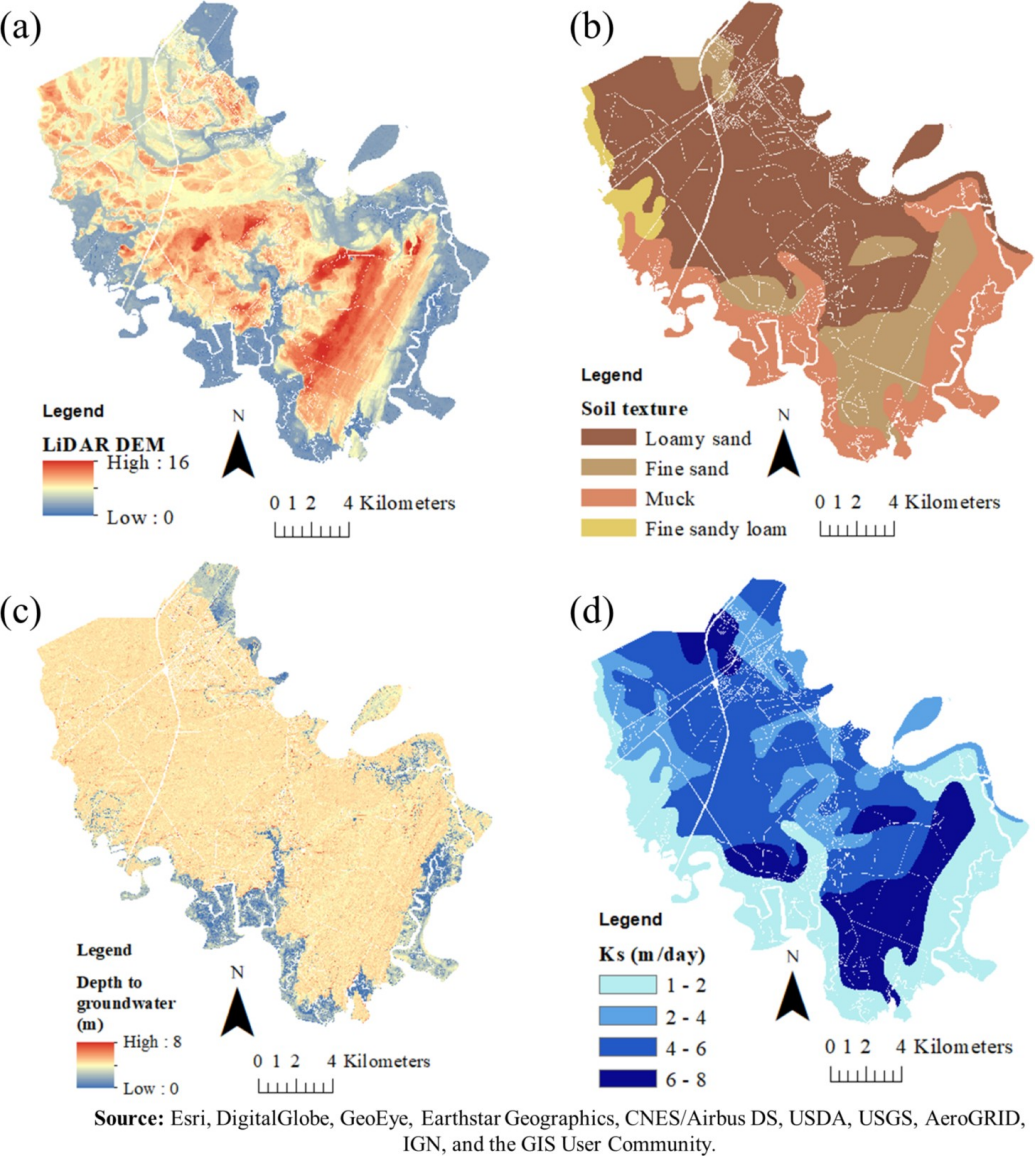
Spatial database of LiDAR Digital Elevation Model (DEM) (a), soil texture (b), depth to groundwater (m) (c), and soil saturated hydraulic conductivity (m/day) (d) of the southern part of Bryan County, Georgia, USA [33].

Most of the Georgia Coastal Plain has very low topographic relief. The LiDAR DEM ranges from 0 m adjacent to intercoastal rivers and tidally influenced marsh areas to a maximum value of 16 m (North American_1983 datum) in the southern or lower part of the county (Fig 2A). The US Army base Fort Stewart functionally splits the county into distinct northern and southern portions (Fig 1). As this analysis was focused on the coastal areas, sufficient data were available in the coastal part of the county, and this bifurcation created problems in developing the groundwater data, as a result this project only examined the southern or lower part of Bryan County.

The geology of the lower Coastal Plain consists of former marine terraces and is composed of unconsolidated sediments [36]. The lower portion of Bryan County is situated on the Pamlico and Princess Anne shoreline deposits which are dominated by fine to very fine quartz sands that are moderately well sorted, with minor amounts of silts and clays. The lower part of the County is located in Tidewater Area and Atlantic Coast Flatwoods physiographic provinces. Both provinces are situated in areas of low topography and very little relief with slopes of less than one percent and shallow depression soils. Atlantic Coast Flatwoods generally have seasonal high water tables and are underlain by marine sands at the surface and loams and/or clays underlie most of the area and restrict the downward movement of water.

The soils in the Tidewater area are mixed sands and clay with poor drainage and the water table typically is close to the surface [37]. Soils are primarily Ellabelle loamy sand and Stilson loamy sand series, with very poor drainage [38] (Fig 2B).

### Groundwater

The surficial aquifer system in Bryan County is composed of Holocene to Pliocene aged sediments and ranges in thickness from over 5 m in the southeastern portion of the county to approximately 43 m in the northern portion of the county [39]. The sediments that comprise the surficial aquifer system are dominated by fine quartz sands with minor occurrences of clays and silts. The aquifer system is in direct contact with the surface and is directly connected with surface water and tidal streams.

**Fig 3:**
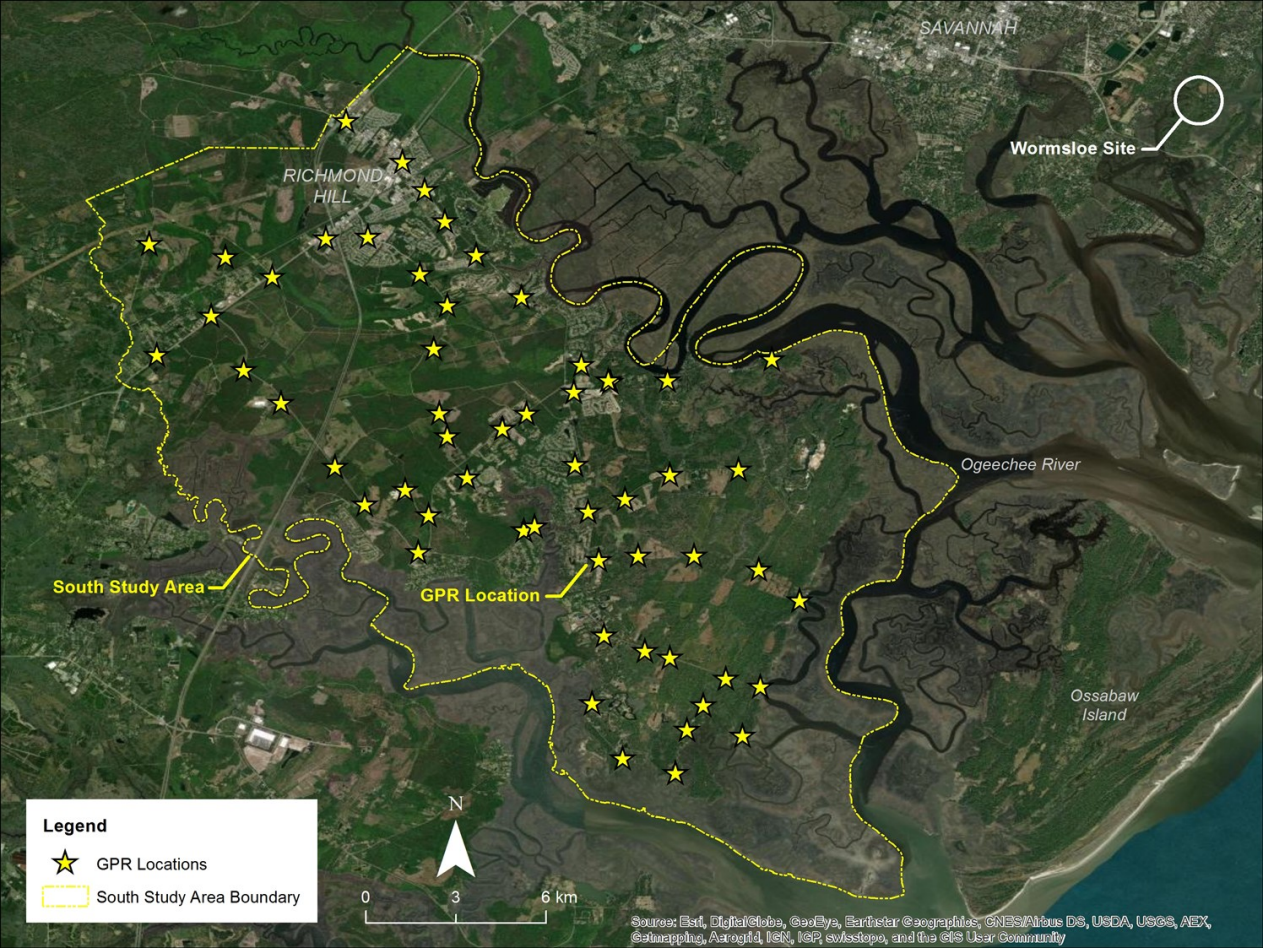
Ground Penetrating Radar (GPR) locations (n = 58) were situated to provide an even spatial distribution and at varying elevations to evaluate topographic influences on the water table. Long-term water level data was used from the Wormsloe Site located to the NE to model seasonal variations in the elevation of the water table [33].

Long term water table data were utilized to construct the initial groundwater model and evaluate seasonal influences in the surficial aquifer from the University of Georgia Center for Research and Education at Wormsloe (CREW) located in Chatham County, Georgia immediately to the northeast of Bryan County (Fig 3). A network of four monitoring wells located at Wormsloe collects continuous water level measurements at a 1-hour frequency. The groundwater data from Wormsloe were utilized to determine the timing and magnitude of the seasonal high water table (SHWT), or the highest water level below which the ground is saturated for 14 consecutive days–and the seasonal low water table (SLWT), or the lowest water level below which the ground is saturated for 14 consecutive days [40].

The seasonal timing of the SHWT was estimated based on the long-term water level data from the background groundwater monitoring site and has been observed to occur during the late winter months when evapotranspiration rates are lowest (December–February). The water table elevation data from the abovementioned location from 2016 to 2018, and LiDAR DEM were used to develop the initial groundwater surface model. Verification of the surface of the water table was performed by using a MALA Ground Penetrating Radar (GPR) system and a controller paired with 160 MHz and 450 MHz antennae. These are shielded antennae that incorporate both transmitter and receiver in one unit at fixed spacings. The GPR data were post-processed for Time-Zero adjustment, spatial interpolation, background removal, 2D spatial filtering, amplitude correction and bandpass filtering.

The depth to groundwater was measured via GPR at fifty-eight locations in Lower Bryan County where accessible and the locations were situated to provide an even spatial distribution and located at varying elevations to evaluate topographic influences (Fig 3). The water table was identified by the change in velocity or a reversal in phase in radar data associated with the interface of unsaturated and water-saturated sands and compared against the preliminary groundwater model [40]. Profiles were calibrated using known groundwater depths at the shallow monitoring wells at the Wormsloe study area and a representative velocity was determined.

GPR profile locations were recorded using a Trimble Geo 7X (Model 88161, Trimble Corp., CA) Global Positioning System (GPS) accurate to within 0.15m. The elevation of the water table was calculated at each GPR location by using the LiDAR surface elevation minus the depth to groundwater as indicated in the GPR data (GW_EL_ = Surface_EL_−GW_Depth_). The relationship between groundwater elevation and LiDAR surface elevation for each GPR location indicated a linear relationship with an *R*^*2*^ of 0.99 as expected in an area of low topographic relief. Therefore, it was assumed that the water table surface strongly follows or mimics the relatively lower elevation surface topography in the area. A water table elevation raster file was generated to model the seasonal low and high-water table elevations using the linear equation and known LiDAR DEM for each pixel of lower Bryan County. Depth to groundwater level raster file for each pixel in the south part of Bryan County was generated from known LiDAR DEM and the calculated water table elevation (Fig 2C). More detailed information about GPR, data collection, and processing can be found in Hodges 2019.

### Modified Soil Topographic Index (STI)

The original STI is derived from widely used Topographic Wetness Index (TWI) [19] that is applicable to low relief topographic setting and incorporates many of the landscape-scale feature indicative of soil saturation (Eq 1) [29]:

$$STI=\text{ln}(\frac{\alpha }{\text{tan}(\beta )K_{sat}D})$$ (1)

where *α* is upslope contributing area per unit contour length (m), tan(*β*) is the local surface topographic slope, *K*_*sat*_ is the mean saturated hydraulic conductivity of the soil (m day ^-1^), and *D* is the soil depth (m). According to the TWI concept, upslope contributing area and drainage (expressed as a topographic slope) of a location affect soil moisture and groundwater level [41]. The higher the TWI value, the wetter the point and the more frequently a point will be saturated to a given level, relative to other points in the same area [42].

In Eq (2), we refined the physical basis of STI in a novel application to septic system vulnerability by coupling it with baseline seasonal high groundwater elevation in the denominator of Eq (1) as:

$$Modified\;STI=\text{ln}(\frac{\alpha }{\text{tan}(\beta )K_{sat}D_{wt}})$$ (2)

where *D*_*wt*_ is depth to the seasonal high water table (m) from ground surface obtained from known LiDAR DEM data and the calculated water table elevation. We used the multiple flow direction D-infinity algorithm [43] to calculate upslope contributing area and slope for each pixel using LiDAR DEM with 1.2 m resolution [44]. Soil saturated hydraulic conductivity was obtained from Web Soil Survey SSURGO soil data [45] for Bryan County and clipped to the parcel layer of the south part of the county (Fig 2D). The modified STI was determined for each pixel within each parcel using ArcGIS 10.3 (ESRI, Redlands, CA, USA) using Eq (2). Using the available data, there was not a good way to determine exactly where is the location of septic system drainfields. Therefore, to determine the modified STI value for each parcel we used Zonal Statistic tool in ArcGIS to calculate the mean of modified STI of pixels within each parcel smaller than 0.04 km^2^ (10 acre) and the mean of modified STI minus one standard deviation of pixels within parcels with the size equal or greater than 0.04 km^2^ (10 acre).

### Septic systems inventory description

We obtained the septic system inventory record for the southern part of Bryan County from the Georgia Department of Public Health (GDPH) online system, which is a publicly accessible database [46]. A total of 3792 septic systems were identified in the study area.

**Fig 4:**
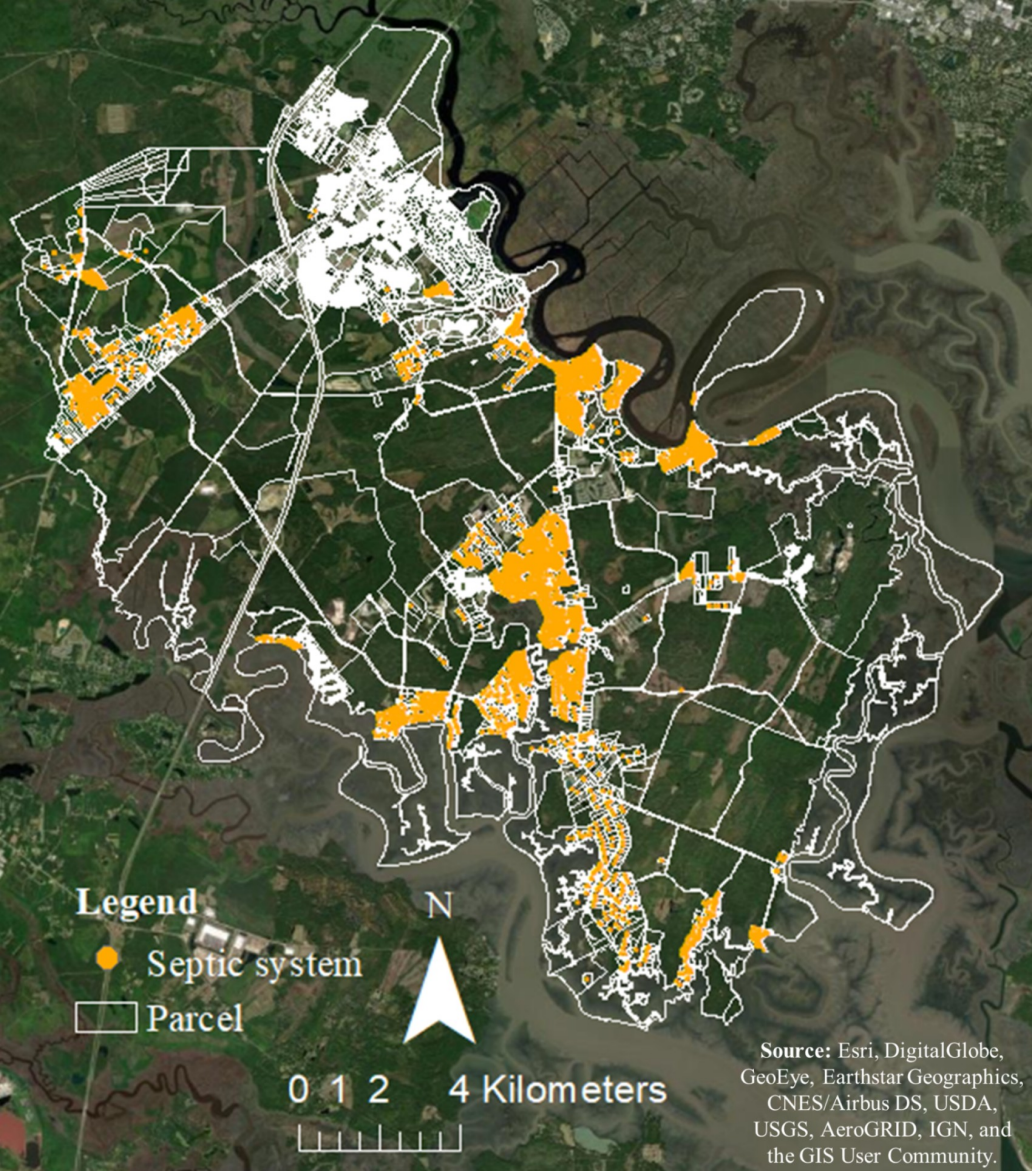
Visual distribution of septic system point features with the available inspection report and property report (n = 3343) within each parcel in the southern part of Bryan County, Georgia [33].

To obtain septic system characteristics (e.g., septic tank capacity, year that septic system was installed, year that structures were built, number of bedrooms, and depth to the water table or restrictive layer) for each septic system point feature from septic system inspection report and property report, we used Joins and Relates feature in ArcGIS to joint data spatially or based on attributes from a table. At the end, the total number of 3343 septic point features with available inspection and characteristics reports was extracted (Fig 4). Then for each septic point feature, the modified STI value calculated from Eq (2) using an ‘Extract Values to Points’ tool in ArcGIS.

In the original inspection report the condition of septic systems was reported in three groups: new (n = 3295), repair (n = 44), and addition (n = 4). ‘Addition’ refers to the installation of a larger septic tank due to the addition to a house, which is required by law. A partial replacement reported as a ‘repair’ system and a full system replacement reported as a ‘new’ system.

The average time required for an septic system to malfunction typically is more than 10 years [47]. Almost 70% of the septic systems that listed as ‘new’ in the original inspection report are more than 10-year old (average = 20 year), which may not have accurately represented the real septic systems condition. To account for an accurate ‘system replacement’, we revised the original inspection report by defining new criteria based on the age of septic system. In addition to septic system ‘repair’ and ‘addition’ (n = 48), if the year that a structure was built significantly predates the installation of septic system (more than 5 years), we assumed the ‘new’ system is replacing an existing failing system. Additionally, if septic systems’s installation year is before the year 2000 those systems are most likely replaced or repaired by the time that the inspection report completed (2014).

In soil that is generally unsuitable for siting a septic system due to conditions such as low permeability, rock formations, or high groundwater elevations septic systems with mounded drainfields may be considered [37]. In these systems, the drainfield is built above the adjacent land grade in a mound of soil that will allow septic effluent to percolate. This increases the separation between the drainfield and the groundwater compared to a drainfield built into the ground. To account for these mounded systems, we divided septic system drainfield type to Mounded (*M*) and Level (*L*) systems based on the state of Georgia a minimum distance separation requirement from the bottom of the drainfield to the groundwater table or other restrictive layer, and we used that requirement to distinguish between the *L* and *M* systems. Prior to 1980, there were no state regulations on installing septic systems, so all systems installed at that time were assumed to be installed as systems with *L* drainfields. In the early ‘80s most GA counties began to require a 30.48 cm (1 foot) separation between the drained field and the groundwater or restrictive layer. Therefore, we defined those systems installed between 1980 and 1997 with less than 30.48 cm of separation from a restrictive layer or water table as systems with *M* drainfields as these systems would require mounds to meet the minimum separation requirements. In 1998, the State of Georgia required that all counties require 60.96 cm (2 feet) separation. Systems installed after 1997 where the depth to restrictive layer or water table is less than 60.96 cm were defined as systems with *M* drainfields (Personal communication with Georgia Department of Public Health) [48].

Septic tank capacity per bedroom was calculated as a tank capacity for each septic system (L) divided by the total number of bedrooms.

### Model development

#### Conditional inference trees model

Recursive binary partitioning methods are a popular statistical tool for regression analysis. These methods provide an alternative to generalized linear models for categorical responses. A Conditional inference trees estimate a regression relationship by binary recursive partitioning in a conditional inference framework. Classification tree models in R (e.g., CART) use internal cross-validation to balance model complexity against the goodness of fit. These models are subjected to overfitting. However, conditional inference trees (e.g., ctree) are unbiased and do not suffer from overfitting. Also, the prediction accuracy of conditional inference trees is equivalent to the prediction accuracy of optimally pruned trees, and no “pruning” or cross-validation is needed [49].

The algorithm involves two steps; in the first step, the algorithm tests the global null hypothesis of independence between any of the input variables and the response variables. The algorithm stops if the null hypothesis cannot be rejected. Otherwise, the algorithm selects the input variable with the strongest association to the response variable. This association is measured by a *p*-value corresponding to a test for the null hypothesis of a single input variable and response.

In the second step, the algorithm implements a permutation test framework to find the optimal binary split in the selected covariate in step one. Then, these two steps repeat recursively [49]. Here, the stop criterion in step one is based on multiplicity *Bonferroni* adjusted *p*-values (*p*-value = 0.05) [50]. A split is implemented when the criterion exceeds the value given by ‘mincriterion’. We implemented mincriterion = 0.95, so the *p*-value must be smaller than 0.05 in order to split nodes. This statistical approach ensures that the right-sized tree is grown without the needs for pruning or cross-validation.

We built a conditional inference tree with ‘septic system’s New or Replace’ as the response (dependent) variable and septic tank capacity per bedroom, modified STI, and septic system drainfield types (*M* or *L*) as the input variables (independent variable). We used the conditional inference tree (ctree) algorithm within the *R* package “partykit” [51]. We used a random sampling of 80% of septic systems with the existing inspection report as a training dataset for the model development (n = 2675) and 20% for the model validation (n = 668).

#### Logistic regression model

We assessed the strength of the relationship between a binary response variable *Y* (0 or 1; septic system’s New or Replace) as a function of input variables *X* through logistic regression model; the effect of the dependent variable is transformed to a probability ratio logarithm (the probability of the event) [52]. In the logistic regression, the predicted values for the dependent variable always lie between 0 and 1 and that can be achieved by applying the following logistic function (Eqs 3 and 4):

$$p(X)=\frac{e^{\left(\beta_{0}+\beta_{1}x_{1}+\cdots +\beta_{n}x_{n}\right)}}{1+e^{\left(\beta_{0}+\beta_{1}x_{1}+\cdots +\beta_{n}x_{n}\right)}}$$ (3)

or

$$p(X)=\frac{1}{1+e^{-(\beta_{0}+\beta_{1}x_{1}+\cdots +\beta_{n}x_{n})}}$$ (4)

where *β*_0_ is the intercept, the *x*_*n*_ independent variables, and *β*_*n*_ their coefficients. We used the maximum likelihood method to estimate *β*_0,_ *β*_1_,…,*β*_*n*_. The continuous probability *p* of the binary dependent variable *y* ranges from 0 to 1. The probability *p* can be transformed to the logit or logistic function as Eq (5):

$$p^{\prime }=\text{ln}(\frac{p}{1-p})$$ (5)

where $\frac{p}{1-p}$ is the likelihood ratio. Theoretically, *p’* can range from minus to plus infinity [53]. In our model, the dependent binary variable is septic system replacement or not (new). The modified STI and septic tank capacity per bedroom were defined as numerical data and drainfield type (mounded or level) a categorical variable. We used *caret* package in *R* statistical software [54].

The *Wald* test was used to assess the statistical significance of each coefficient in the model. The *Wald* test is equal to the ratio of the maximum likelihood estimate of the slope parameter (*β*_*n*_) to an estimate of its standard error [52]. The result is significant if its standard distance from zero is large enough. The logistic regression model indicates the relationship between the most significant explanatory variables and the response variables. However, the analysis cannot examine the threshold of each predictor at which septic system replacement occurred.

#### Model accuracy

We evaluated the models’ performance with two accuracy measures; the overall accuracy for confusion matrix and the area under the Receiver Operating Characteristic (ROC) curve. For each model (conditional inference model and logistic regression) we calculated the overall accuracy measure for the confusion matrix, a table that contrasts predicted vs. observed classifications, for training and validation datasets. Then we calculated the accuracy measure for the confusion matrix for the validation dataset to assess the ability of the model to predict septic systems’ replacement rate.

Overall accuracy for the models is calculated from the confusion matrix, and it is the sum of the true positive and true negative of classifier divided by a total number of observations on training or validation dataset. Values close to one represents the ability of the model to predict a binary response more accurately [52]. The area under the curve (AUC) evaluates true positive (sensitivity) and false positive (specificity) of the model and ranges from 0 to 1. ROC curve is used to estimate the accuracy of a continuous measurement for predicting binary response. In the ROC curve sensitivity is plotted on the *y*-axis and 1-specificity on the *x*-axis. Values higher than 0.5 indicating model performed better than would be expected from chance alone and a value of 1 specifying perfect agreement [55]. The probabilistic interpretation of AUC is that if a true positive and a false positive classifier are chosen randomly, AUC indicates the probability that positive classifier outranks the negative classifier. For calculating AUC we used *ROCR* package in *R* statistical software [56].

## Results

### Modified Soil Topographic Index (STI) and septic system characteristics

**Fig 5:**
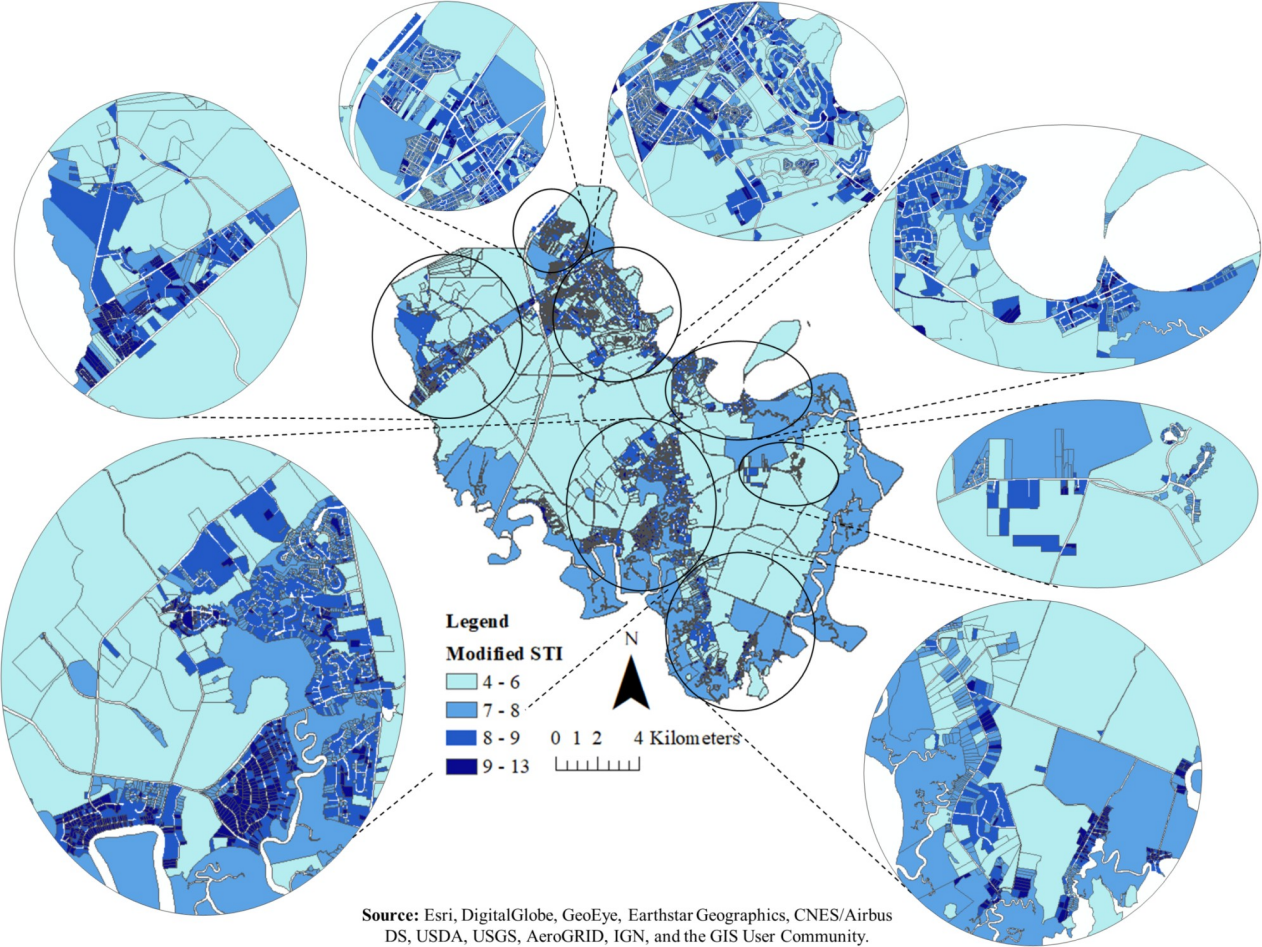
The map of modified STI for all parcels in the southern part of Bryan County. The modified STI for parcels smaller than 0.04 km^2^ (10 acres) was calculated as a mean of the modified STI for each pixel within that parcel and for parcels equal or greater than 0.04 km^2^ (10 acres) was calculated as a mean of the modified STI minus one standard deviation of each pixel within that parcel. Small numbers represent dryer soil conditions and large numbers represent wetter soil conditions.

The calculated modified STI for all parcels has a normal distribution and ranges from 5 (dryer) to 13 (wetter) (Fig 5). In our model, we implemented the modified STI values for parcels with septic system. Parcels with a high wetness index occur where there is a combination of low slope and high flow accumulation [57] and therefore, it may indicate locations with higher septic system malfunction.

**Fig 6:**
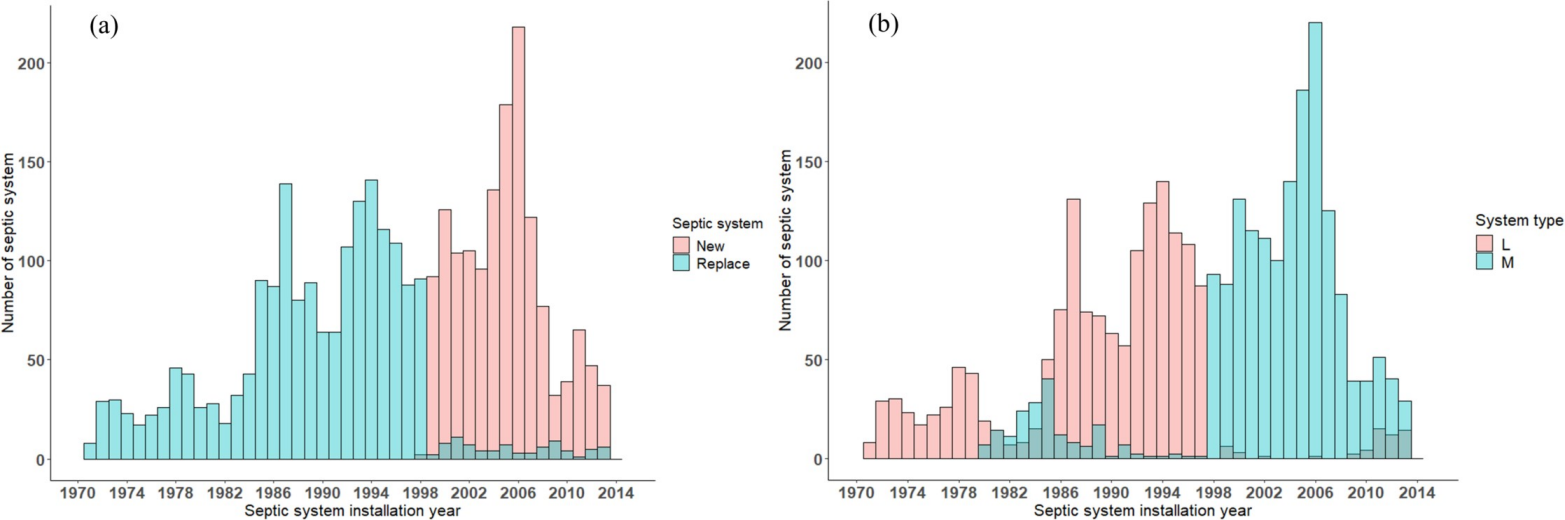
Bar graphs of the number of septic systems’ New or Replace (a), and Mounded (*M*) vs. Level (*L*) septic system drainfields (b), as a function of septic system installation year for revised inspection report in the southern part of Bryan County, Georgia.

Based on the methodology described in the Materials and Methods section, the total numbers of ‘new’ systems in the revised version of the inspection report was 1479 and the total number of ‘replace’ systems including partial repair, addition, and full replacement was 1864 (Fig 6A).

As described above, we followed the state of Georgia minimum distance of separation requirement to divide septic system drainfield type into *M* and *L* systems. Prior to 1980 all 244 septic systems were installed as systems with *L* drainfields (Fig 6B). From 1980 to 1997 the total number of systems installed with *M* and *L* systems was 183 and 1268, respectively. With doubling minimum distance of separation requirement after 1998 in Georgia, most of the systems at the southern part of Bryan County were installed with *M* drainfields (*M* = 1590) and only 58 systems were installed with *L* drainfields (Fig 6B). Septic tank capacity per bedroom ranged from 541 to 3785 (L) with an average of 1257 (L) per bedroom.

### Conditional inference trees model

**Fig 7:**
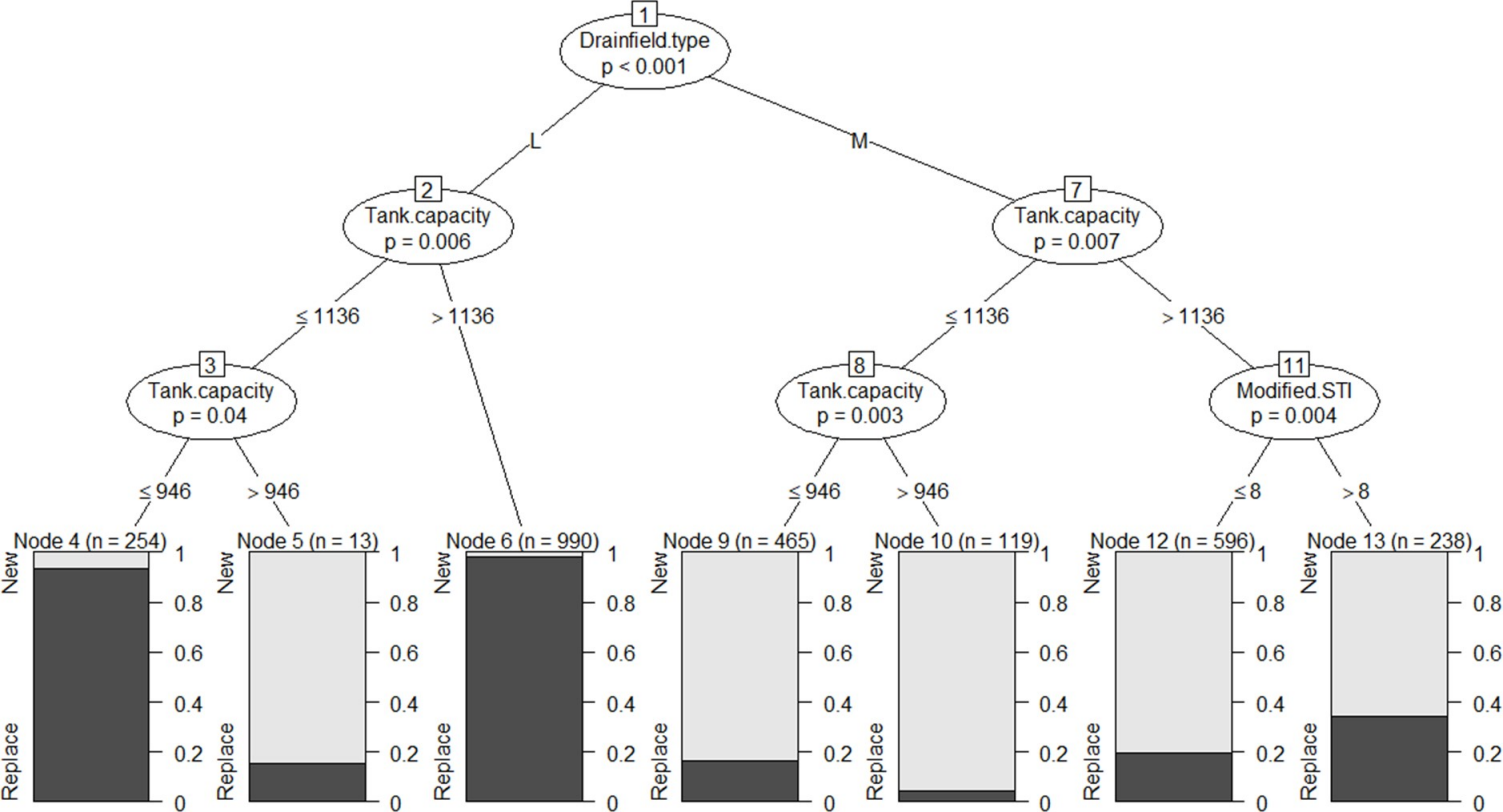
Classification inference tree model based on the training data set (n = 2675). Values and letters (*M* = Mounded and *L* = Level) on lines connecting input variables indicate splitting criteria (e.g., if it is noted M all septic systems with Mounded drainfield were placed in the group to the right on the branch, otherwise there were placed on the branch to the left). Numbers in boxes attached to input variables show the node number. “n” above the terminal nodes indicates the number of septic systems classified in that node. “*p”* represents the *p*-value for splitting the nodes (here *p*-value < 0.05). Tank capacity was calculated as litter per bedroom. Bar charts illustrate the proportion of septic systems Replace (black) or New (light gray) in that node.

We illustrate the relationship between the response variable (septic systems Replace or New) and input variables with a dichotomous tree diagram for the training dataset (Fig 7).

Each oval node represents the splitting variable and branches that connect nodes. Each rectangular box in the conditional classification tree represents a terminal node that represents the final groups. The “n” in each rectangular box represents the sample size. The *ctree* model produced a tree with six splits and seven terminal nodes (Fig 7). The first oval node, septic system drainfield type, indicates a strong association with the response variable (septic system Replace or New) with the *p*-value < 0.001. Following the right branch of the tree, systems with mounded drainfield (*M*), septic tank capacity per bedroom (Tank.capacity) is the splitting node which indicates this variable has a strong association (*p*-value = 0.007) with the response variable (Fig 7). Continuing down on the right branch of node 7, the modified STI is a significant variable in predicting the septic system replacement rate. The septic systems with tank capacity per bedroom greater than 1136 L (300 US gallon per bedroom) and a modified STI value greater than 8 (right branch of node 11), have the highest replacement rate of 34% for *M* systems (n = 238 and *p*-value = 0.004). However, those septic systems with the same tank capacity per bedroom but a modified STI equal or smaller than 8 have only a 20% replacement rate (n = 596 and *p*-value = 0.004) (Fig 7). On the left branch of node 7 (tank capacity per bedroom equal or less than 1136 L) capacity per bedroom again became the splitting variable. The lowest replacement rate of 4% was observed in septic systems with the tank capacity per bedroom of more than 1136 L (300 US gallon per bedroom). On node 8, septic systems with smaller capacity per bedroom equal or less than 946 L (250 US gallon per bedroom) has a replacement rate of about 16% (n = 465 and *p*-value = 0.003) (Fig 7).

Similarly, following the left branch of the tree (Fig 7), systems with *L* drainfield, tank capacity per bedroom (Tank.capacity) is the splitting variable (*p*-value = 0.006). Although systems with *L*, the tank capacity per bedroom is the only significant variable for predicting the replacement rate. Following the right branch of node 2, the septic systems with tank capacity per bedroom greater than 1136 L (300 US gallon per bedroom) have the highest replacement rate of almost 98% (n = 990, *p*-value = 0.006). Moving further down the branch under node 3, again tank capacity per bedroom became the splitting variable, and systems with *L* drainfield with capacity per bedroom less than or equal to 946 L (250 US gallon per bedroom) results in replacement rate of 93% (n = 254, *p*-value = 0.04) as compared to the *L* drainfield with capacity per bedroom of 1136 L (300 US gallon per bedroom) (replacement rate of 15%, n = 13).

### Logistic regression model

The logistic regression analysis showed the nonlinear relationships between the independent variables and septic system replacement probability (response variable). The model regression coefficients, standard errors of the slope coefficients, *Wald* test, and significance levels (*p*-values) are presented in Table 1. The overall accuracy of the logistic linear regression model for the training data set was 88%. The results showed that all three independent variables are significant in predicting septic system replacement probability, with the *p*-value < 0.05 for the modified STI, and *p*-value < 0.001 for septic system drainfield type (Drainfield.type) and septic tank capacity per bedroom (Tank.capacity) (Table 1). The Wald test values showed that septic system drainfield type was statistically the most significant predictor of systems replacement rate, followed by septic tank capacity per bedroom (Tank.capacity) and the modified STI (Table 1).

**Table 1:** The output of the logistic regression model for training dataset: Coefficients (*β*_*n*_), standard error, *Wald* statistics, and *p*-value.

	Variables	Coefficient (*β*)	Standard error	Wald	*p*-value
**Model 1**	Drainfield.type	-4.66	0.16	827.73	<0.0001
**Model 2**	Modified.STI	0.17	0.05	11.20	<0.001
**Model 3**	Tank.capacity	4 × 10^−4^	1× 10^−4^	13.02	0.001
**Model 4**	Drainfield.type	-4.66	0.16	790.77	<0.001
	Modified.STI	0.12	0.08	2.71	0.1
**Model 5**	Drainfield.type	-4.67	0.16	793.28	<0.0001
	Tank.capacity	5 × 10^−4^	2 × 10^−4^	6.25	0.002
**Model 6**	Modified.STI	0.18	0.05	12.09	0.0005
	Tank.capacity	4 × 10^−4^	1 × 10^−4^	11.61	0.001
**Model 7 (final)**	Drainfield.type	-4.66	0.17	790.40	<0.0001
	Modified.STI	0.13	0.08	3.03	< 0.05
	Tank.capacity	5 × 10^−4^	2 × 10^−4^	8.73	<0.001

The *p*-value < 0.05 is significant.

The intercept for our final model was 1.53. Based on the model 7 in Table 1, coefficients and Eq 4 the probability of septic system replacement rate can be calculated for each parcel with septic system as Eq (6):

$$p=\frac{1}{(1+e^{-(1.53+0.13[Modified.STI]+0.0005[Tank.capacity]-4.66[Drainfield.type])}}$$ (6)

### Model validation

**Fig 8:**
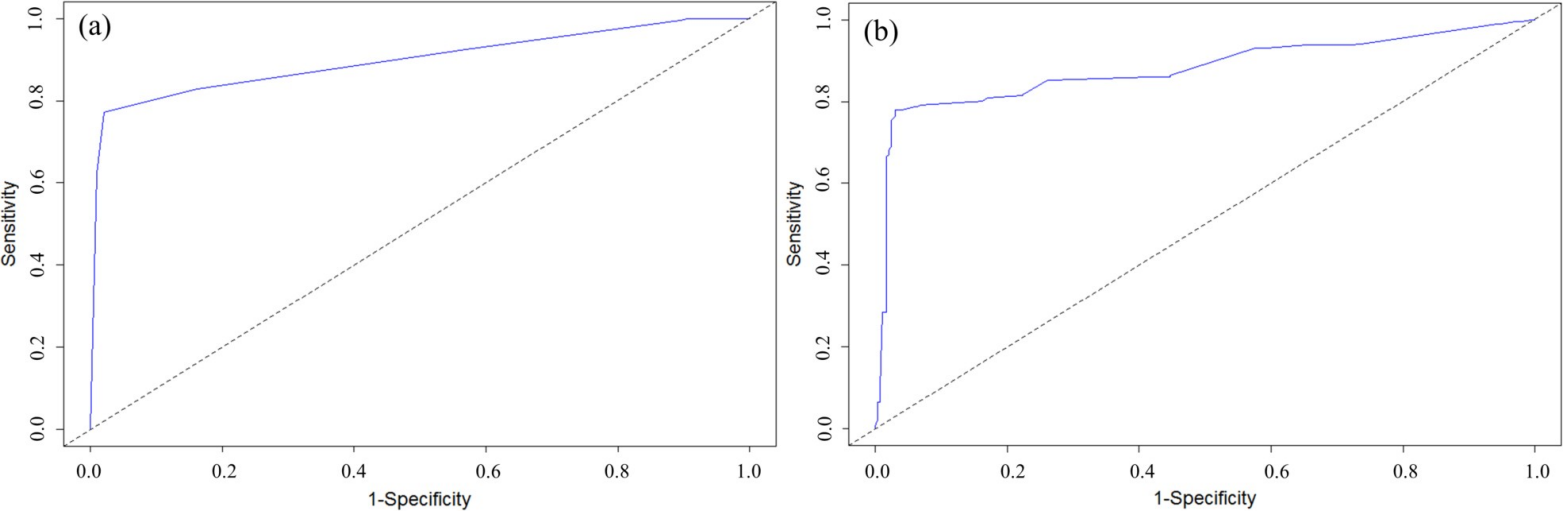
Receiver Operating Curve (ROC) for the validation dataset for classification inference tree model (a), and logistic regression (model 7) (b). Sensitivity (y-axis) describes the proportion of classifiers that are correctly predicted and specificity is the proportion of classifiers that are falsely predicted. AUC is the area under the receiver operating curve. A dash black line is a reference line.

To explore both conditional inference tree and logistic regression models’ performance, the AUCs were quantitatively compared for validation datasets (Fig 8A and 8B). The model with the highest AUC is considered optimal (Table 2). The AUC is 0.90 and 0.88 for conditional inference tree and logistic regression, respectively. In general, AUC between 0.80 to 0.90 is considered an excellent model fit [52].

**Table 2:** Overall accuracy, true positive (sensitivity), and Area Under the Curve (AUC) values for conditional inference tree and logistic regression models for validation dataset.

Classification model	Overall accuracy ^a^	True Positive ^a^ (sensitivity)	AUC
**Conditional inference tree**	0.87	0.80	0.90
**Logistic regression (model 7)**	0.86	0.76	0.88

^a^ Overall accuracy and True Positive were obtained from the confusion matrix for the validation dataset.

Overall accuracy was calculated from the confusion matrix, which is the sum of true positive and true negative divided by total validation data point (here n = 668). The overall rate of correct classification for validation dataset was 87% for conditional inference tree and 86% for logistic regression model showing low model misclassification error rate, with lower misclassification error rate for the conditional inference tree model (12%) than for the logistic regression model (14%). The correct classification or true positive (sensitivity) is the group of septic systems that actually experienced replacement. True positive is 80% and 76% for the conditional inference tree model and logistic regression model, respectively (Table 2). Overall, the results of the model validation indicate that both conditional inference and logistic regression models can be used to predict the septic system replacement rate. However, the conditional inference model indicates a slightly better prediction of the actual replacement rate than the logistic regression model with a higher true positive rate of 0.80 in comparison with 0.78 (Table 2).

**Fig 9:**
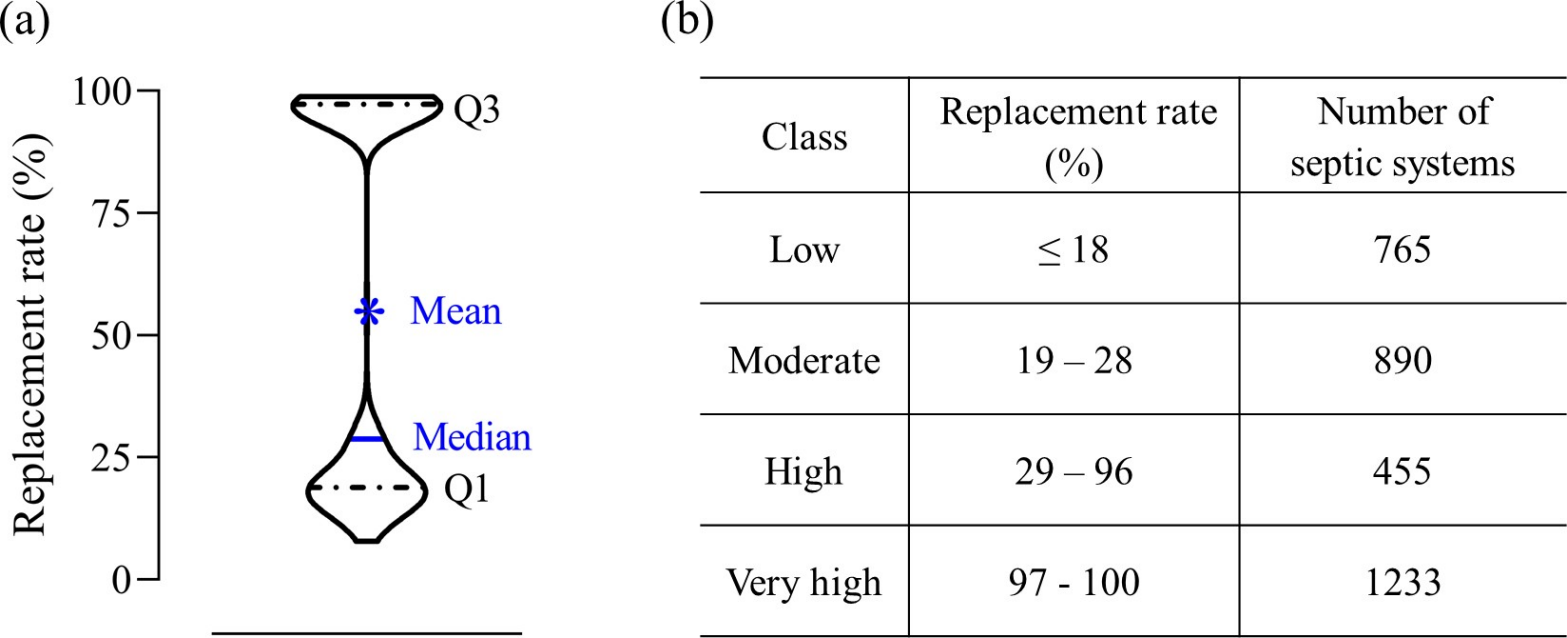
Distribution of septic systems replacement rate (%) (a), and class, calculated probability replacement rate (%), and the number of septic systems in each class (b) in the southern part of Bryan County, GA. Q1 represents the first quartile, and Q3 represents the third quartile.

**Fig 10:**
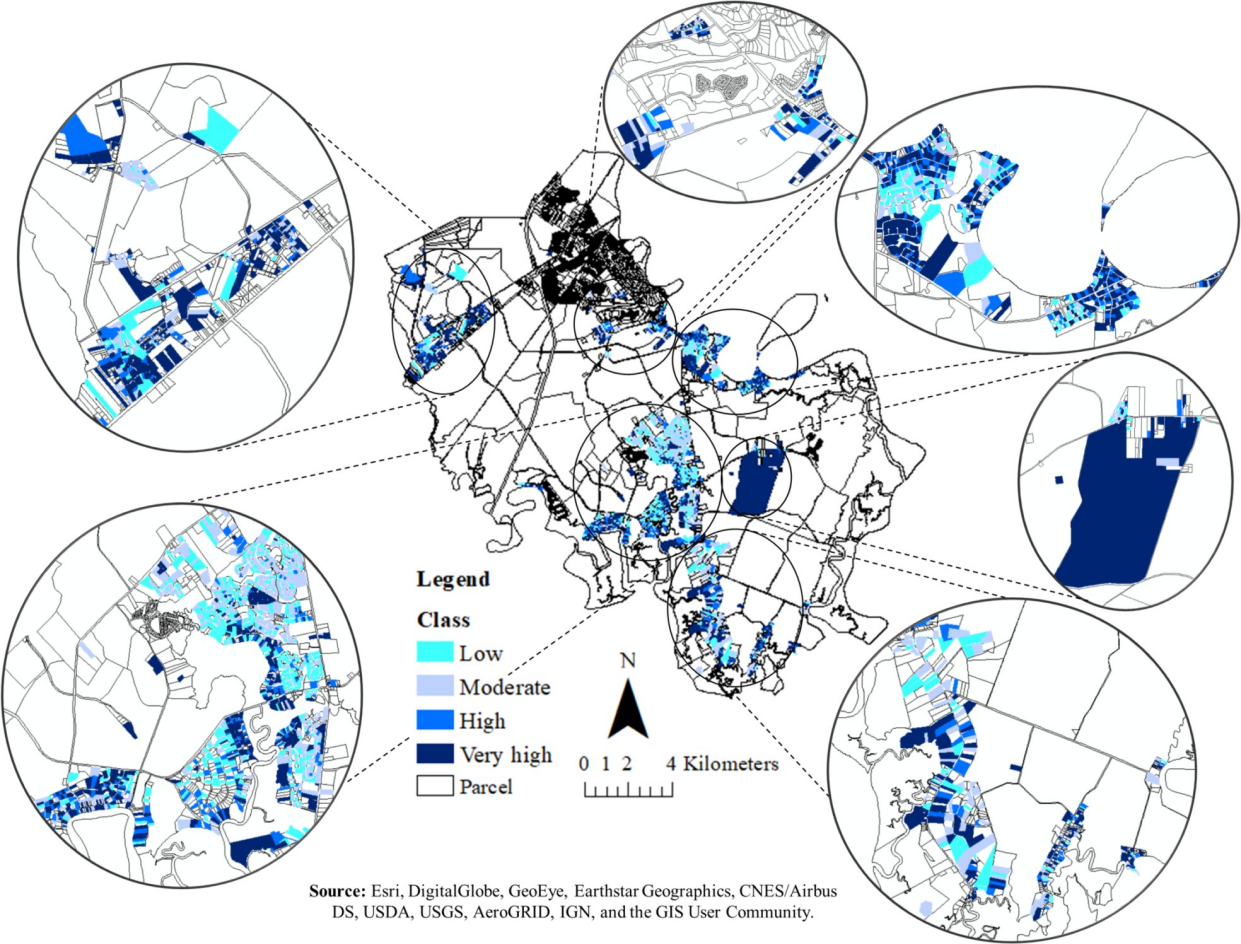
Classification of septic system replacement rate for the southern part of the Bryan County using classification inference tree and logistic regression.

To visualize this, because the AUC and true positive values for both models are not significantly different, averaging the output probabilities is an effective ensemble method to get the final septic system replacement probability for each parcel. Then the calculated probability for system replacement was classified using natural breaks as low (≤ 18%, first quartile), moderate (19% - 28%, median), high (29% - 96%, third quartile), and very high (97% - 100%, maximum) (Fig 9A and 9B) and presented in Fig 10.

## Discussion

### Model performance

We used classification inference and logistic regression models to predict the probability of septic system replacement. Both models have high AUC values (0.90 and 0.88 for classification tree and logistic regression, respectively) (Table 2). These high AUC values show that both models can predict septic system replacement rate in our study. However, the classification tree model demonstrated the threshold of each input variable (drainfield type, modified STI, and septic tank capacity per bedroom) that significantly affect predictions of the septic system replacement. The data for the properties-built year were available in the original inspection report, but it was significantly correlated with the septic system age (*r* = 0.72, *p*-value < 0.01). It can be hypothesized that older structures have older septic systems. However, in the original report more than 70% of systems 10 years old or older were listed as new. Hence, we used the age of systems as a key factor to modify their condition in the original inspection report Therefore, we did not include age as an independent variable. Correlation analysis showed that age of septic systems is significantly correlated only with the modified STI, but the correlation was not strong enough (*r* = 0.13, *p*-value < 0.01) to conclude that the older systems are necessarily sited in more unsuitable moisture conditions.

The results of the classification inference tree indicate that septic systems with a level drainfield (*L*) have a higher average replacement rate of 96% compared with the mounded drainfield (*M*) with an average replacement rate of 20% (Fig 7). This likely indicates the importance of septic system siting criteria such as the minimum vertical separation distance between the bottom of trenches and seasonal high water table or restrictive layer. Systems with an ‘L’ drainfield and large capacity of the septic tank per bedroom (> 1136 L) had the highest replacement rate (98%) (terminal Node 6). Of these systems, 973 systems out of 990 systems (98%) were installed before the year 1998 (average age of 25 years). From those, 17% of systems (n = 167) were installed before year 1980 when there was no state regulation on minimum requirement in vertical separation, and 83% (n = 806) were installed between 1980 and 1998, when a 30.48 cm minimum separation between the bottom of trenches and seasonal high water table became required. Despite large septic tank capacity per bedroom, this high replacement rate was expected, likely because of insufficient mandatory horizontal separation distance. The same is true for the septic systems with tank capacity of equal or smaller 946 L (250 US gallon per bedroom) almost 93% of systems were installed before mandatory horizontal separation distance of 60.93 cm (2 ft) (Node 3, terminal Node 4). Furthermore, these systems have smaller tank capacity per bedroom compared with systems in Node 5, causing the higher replacement rate due to higher effluent loads than tank design capacity. In contrast, of the systems with ‘L’ drainfield and tank capacity of 1136 L (> 946 L) only 2 out of 13 systems were installed before 1998, and those have only a 15% replacement rate.

We observed the lowest replacement rate of 4% in septic systems with mounded drainfield and septic tank capacity per bedroom of 1136 L (300 US gallon per bedroom) (Fig 7). Again, septic systems that fall in this terminal node (Node 10) are newer (average age of 6 years) than those in any other terminal nodes in the ‘*M*’ drainfield branch. This indicates that septic tank capacity per bedroom of 1136 L (300 US gallon per bedroom) approximates a threshold tank capacity for newer systems in our study to see the lowest replacement rate. Mainly, septic systems with ‘*M*’ drainfield, tank capacity per bedroom greater than 1136 L (300 US gallon per bedroom), and the modified STI > 8 have higher replacement rates (average of 34%) in comparison with systems with modified STI ≤ 8 (19%). Parcels with modified STI > 8, represent wetter conditions than modified STI ≤ 8, and this wetness may decrease the septic system functionality. Even for recently installed systems, this indicates the significance of site hydrological characteristics (modified STI) in predicting systems functionality. Cox et al., 2019 also showed that changing hydrological characteristics (i.e., rising groundwater tables) in coastal aquifers in southern Rhode Island, may reduce OWTS functionality, threatening coastal ecosystems and drinking water aquifers with nutrient and pathogen pollution [58].

A larger replacement rate (16%) was expected for septic systems with mounded drainfield and tank capacity of 946 L (250 US gallon per bedroom) or less (Node 8, terminal Node 9) than systems with larger tank capacity per bedroom (Node 8, terminal Node 10) (Fig 7). Almost 61% of septic systems that experienced replacement in terminal Node 9, were installed after 1998. Despite the fact that these are almost all newer systems, inadequate tank capacity and/or higher effluent loads than design capacity have significant impacts on the likelihood of replacement.

The results of the final generalized linear regression model (Table 1, model 7) suggest that drainfield type, septic tank capacity per bedroom, and the modified STI each exert a significant influence on predicting septic systems replacement rate. Given the log link function, a unit change in independent variables (*x*) has a multiplicative effect on the response variable (*Y*). Where *e*^*β*^ > 1 the variable increases the expectation of response variable and *e*^*β*^ < 1 decreases the expectation [17]. For instance, a unit increase in the modified STI increases the replacement rate by a factor of 1.143 = *exp* (0.1338), where a unit increase in septic tank capacity per bedroom has a factor of 1.000 = *exp* (0.0007), indicating that increasing septic tank capacity has minimal effect on increasing systems replacement rate.

### Policy, planning, and management implications

Once regarded as a temporary solution for wastewater management in areas where centralized sewage treatment would eventually be installed, septic systems are now recognized as a permanent component of community infrastructure in many areas [1]. While these systems can provide effective long-term wastewater treatment when property installed and maintained, they can cause significant problems for public health and environmental quality when they are not. To date, planning and regulatory efforts have not dealt with the long-term functionality of these systems.

Few communities actively monitor the functioning of systems or mandate regular system maintenance. Recognizing that poorly sited and improperly designed systems can cause significant problems, septic system research and regulation has focused on optimizing their performance through system design and siting. In Georgia, for instance, the state has implemented regulatory measures increasing the required distance between the septic drainfield and the groundwater table or impermeable layers, and requiring dual-chambered septic tanks that help prevent wastewater solids from clogging the drainfield [1]. While changes such as these have been successful in improving septic system functionality and reducing failures, the present regulatory and policy scheme is ultimately limited in its ability to address the long-term impacts of septic systems as they primarily look at the initial installation. For instance, Cox et al., 2020 concluded that revising the septic system regulatory permitting process (i.e., inadequate separation distance from the infiltrative surface of the drainfield to the seasonal high water table) in southern Rhode Island may help protect coastal drinking and surface water resources [59].

Developing better local data on the location and condition of septic system promises to make management and regulation of installed septic systems more feasible and beneficial. The methodology described here provides the ability to assess the functionality of septic systems over time and at scale potentially allows for a much more sophisticated and effective septic system management. For instance, systems might be prioritized for additional attention if it is deemed highly likely to fail, or additional siting requirements could be instituted to account for variance in the STI. In addition, this analysis could be used to target outreach efforts regarding septic system maintenance and operation, or to direct water quality monitoring efforts based on likely concentrations.

In addition to improving septic system maintenance and regulation, this methodology can also benefit future planning and development decisions in coastal Georgia and other coastal areas with similar geophysical characteristics. The use of the digitized and geo-located septic records in this process allows the analysis of how hydrological factors interact with other variables such as tank capacity per bedroom, drainfield type, housing characteristics, system age, and other elements to influence system performance. In addition, this process allows planners and regulators to examine the impacts of septic systems at landscape or watershed scales. Past efforts to report on the impacts of septic systems to environmental quality and public health have relied on testing individual systems to identify failures and extrapolate the effects of those failures across larger areas [1]. This process allows planners and resource managers to examine septic systems in place and in the aggregate, which creates significant opportunities to better understand their impact on the environment and public health. Moreover, this approach is transferable to countries relying on septic systems or other on-site sewage systems (e.g., cesspool) for their wastewater treatment where limited access to clean water and sanitation causes significant public health issues [14, 15, 60].

We encountered some limitations in this project. First, the septic system location data identified parcels with septic systems, and the drainfields’ location was not marked within the parcel. As a result, the parcel centroid was used as the drainfield location. For most parcels, this was not a significant issue because they are smaller than 0.04 km^2^ (4 acres), which means the actual location was nearby. However, for larger parcels, this is more problematic, though this only represents 2.5% of the systems assessed, and the efforts to create a generalized assessment of the parcel ameliorated some of the impacts of this limitation. Another limitation of note is the difficulty translating the condition and maintenance data fields into repair and replacement data as many of the systems reported to “new” even though it is clear that these are actually new installation of replacement systems. The process used to identify replacement systems based on the year the associated structure was constructed resolved much of this issue, but it is likely that some number of system failures and replacements were missed as a result. Despite these constraints, the overall results of the analysis provided valuable information that improved the understanding of the hydrological factors associated with septic system vulnerability in coastal areas.

## Conclusions

By demonstrating how modified STI values can be related to the vulnerability of septic system failure, we create a valuable metric that could radically improve the way septic systems are managed. Current septic system siting and management decisions are based on a single assessment of site conditions at the time of installation. There are no dynamic variables that account for changes in soil moisture due to changes in the groundwater table or other climatic changes. This novel application of the STI incorporating the baseline seasonal high groundwater elevation and known septic system specifications to create a GIS-based framework for septic system vulnerability using classification inference tree and generalized logistic regression models creates a metric that can be used to evaluate how a site’s suitability for a septic system changes over time. This process is based on a newly developed Bryan County Health Department’s database of septic system characteristics such as septic tank capacity, year the system was installed, year that the attached structures were built, number of bedrooms in those structures, and depth to the water table or restrictive layer. The results confirmed our hypothesis that both the modified STI and septic system specifications, such as tank capacity per bedroom and drainfield type explained most of the variance in septic system repair and replacement. The model validation outputs showed that both models can use to predict septic system replacement rate. Although, the conditional inference tree provided a better prediction of the replacement rate compared to the logistic regression model with a higher true positive rate.

Overall, we found that septic system drainfield type (level vs. mounded) is a significant variable in predicting septic systems vulnerability. Systems with a *L* drainfield have a higher average replacement rate (96%) than systems with *M* drainfield (20%). This implies the importance of horizontal separation distance between the bottom of drainfields and seasonal high groundwater or other restrictive layers. In addition, for septic systems with *L* drainfields, tank capacity per bedroom was significant variable in predicting the replacement rate. For systems with *M* drainfields, tank capacity per bedroom was a significant variable in predicting failure, although, the soil hydrological condition of systems with a tank capacity of greater than 1136 L (300 US gallon per bedroom) was also a significant variable in predicting septic systems functionality. In short, we conclude that for recently installed systems designed with appropriate tank capacity per bedroom, site condition plays an important role in septic system viability.

The septic system vulnerability data and maps will be valuable tools to aid decision-making with respect to existing system maintenance and operation, as well as future site selection, design specifications, and even future land use planning. Also, the modeling tool may also serve as the basis of septic system policy and management decisions at a larger scale such as at county level and watersheds scales.

Future investigations that are possible with this methodology include improving our understanding of septic system viability social, economic, and demographic variable by lining additional data sources such as census data or a social vulnerability index with our model. Furthermore, coastal areas are particularly vulnerable to climate change, specifically the hydrological changes that cause by sea-level rise. Future research that integrates plausible sea-level rise scenarios will help to assess the impacts of sea-level rise on groundwater elevation, soil saturation, and septic system performance and viability.
